# Immediate and Sustained Resolution of Persistent Primary Monosymptomatic Enuresis Following Fascial Counterstrain Therapy: A Case Series

**DOI:** 10.7759/cureus.104063

**Published:** 2026-02-22

**Authors:** Maria DelGiorno

**Affiliations:** 1 Pediatric and Adult Osteopathic Manual Therapy, Wellspring Release LLC, North Potomac, USA

**Keywords:** adolescent bedwetting, adolescent urology, bedwetting alarm, fascial counterstrain, interstitial inflammation, manual therapy, monosymptomatic enuresis, osteopathic manipulative treatment (omt), primary nocturnal enuresis, urogenital fascia

## Abstract

Primary monosymptomatic enuresis (ME) that persists into adolescence presents significant physical and psychosocial challenges and is often resistant to conventional therapies. This case series describes three adolescents (aged 10, 12, and 15 years) with persistent, nightly ME who experienced immediate and sustained resolution of symptoms following Fascial Counterstrain (FCS) therapy. FCS is a multi-system manual therapy that applies an indirect osteopathic approach to restricted anatomical dysfunction and is well tolerated. In this study, FCS techniques associated with urogenital dysfunction were identified and treated in three adolescent cases. In each case, cessation of bedwetting occurred within 24 hours of the FCS session. Long-term follow-up at 10 to 24 months demonstrated continued resolution without the need for further treatment. These findings indicate that urogenital fascial restrictions may contribute to ME in some patients; however, further controlled research is required to validate these observations and explore the underlying physiological mechanisms.

## Introduction

Adolescent primary monosymptomatic enuresis (ME), defined as bedwetting since birth without daytime symptoms, represents a significant and often underreported clinical challenge in adolescence and adulthood. It is estimated that, at a given time, approximately six million individuals over age 10 in the United States suffer from ME [1]. Adolescent cases are typically severe, with nightly or almost nightly episodes, as mild bedwetting (less than three times per week) is more likely to have resolved by this age [2]. Approximately 2%-4% of adults remain enuretic, as the rate of spontaneous remission declines over time [3]. Despite reports confirming a significant psychosocial burden, approximately 70% of these adolescent and adult patients no longer seek medical advice, often due to a perceived lack of curative options [3].

This case series explores a novel treatment for ME using Fascial Counterstrain (FCS) manual therapy to address potential physical restrictions in urogenital structures. Based on the mainstream indirect osteopathic technique of Strain and Counterstrain developed by Dr. Lawrence Jones, FCS has evolved into a multi-system technique that anatomically correlates not only with musculoskeletal tissues but also with visceral, vascular, and neuromeningeal tissues [4]. Although the physiological mechanism of action of FCS remains unknown, it has recently been theorized that the treatment promotes drainage of pro-inflammatory cytokines (IL-1b, IL-6, and TNF-alpha) into regional lymphatics by means of local tissue decompression [5]. The objective of this report is to document the immediate and sustained clinical outcomes of FCS in three adolescents with ME, offering a potential new avenue for treatment in this underserved population.

## Case presentation

FCS treatment overview

**Figure 1:**
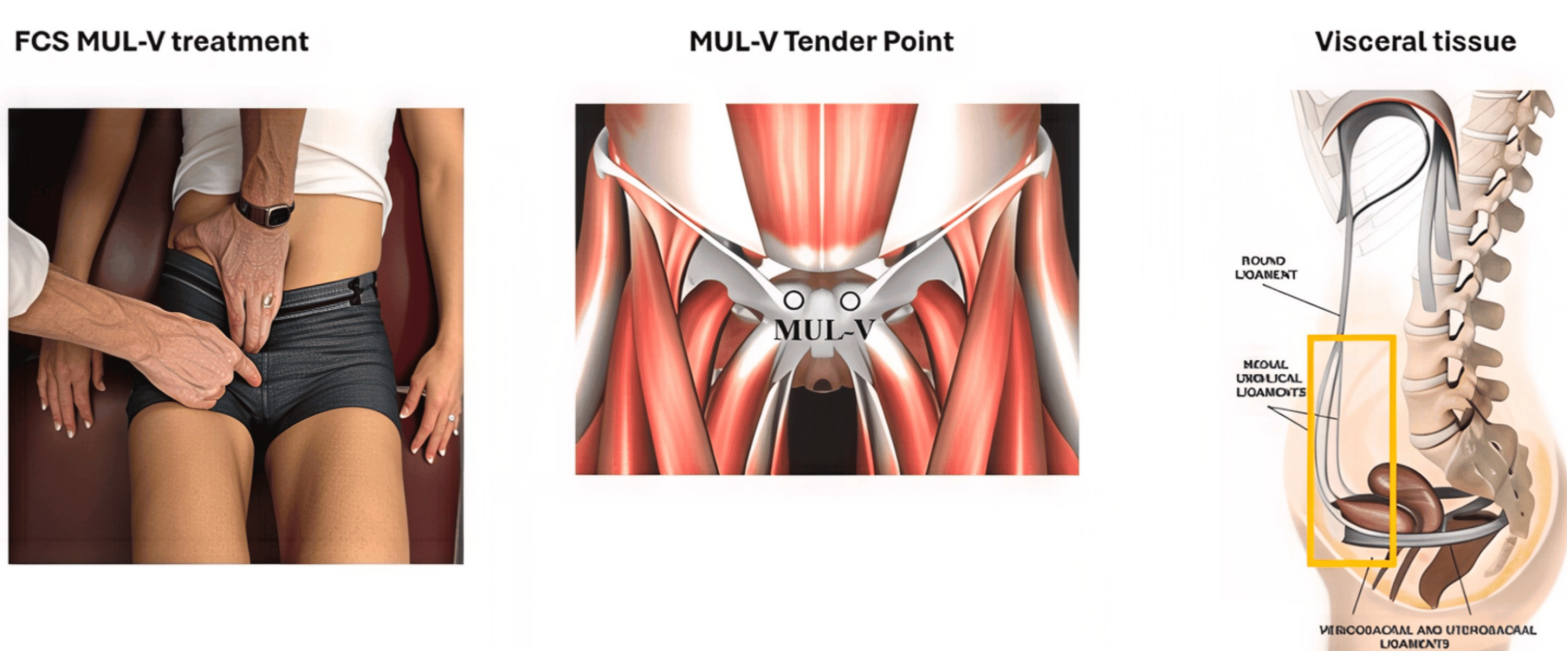
Medial Umbilical Ligaments-FCS Visceral (MUL-V) Technique Tender Point: Over the superomedial pubic bone, adjacent to the pubic symphysis. Treatment position: Supine, hips and knees flexed. Fascial glide: Glide the anterior abdominal wall in an inferolateral then posterior direction (using your fingertips). Symptoms: Sacral pain, bladder dysfunction, hamstring hypertonicity. Image reproduced from Tuckey [4] under the terms of the Creative Commons Attribution (CC BY) License.

The FCS treatments in this series were performed by a physician trained in the technique. From a curriculum of over 900 anatomically correlated techniques, the provider focused on assessing approximately 50 that could potentially impact the urogenital system. The provider performed a detailed physical examination to identify an active or painful tender point (TP), applied a manual glide held for approximately 30 seconds, reassessed to confirm that the TP was resolved, and repeated the assessment and treatment until all active TPs were resolved within the allotted time. Figure 1 presents an example of the medial umbilical ligament FCS visceral technique, which commonly required release.

Case 1

A 10-year-old male presented with nightly bedwetting since birth. His history included a tonsillectomy and adenoidectomy at age three. He had not previously attempted medications or bedwetting alarms. Review of his medical record since birth revealed no documentation that he or his mother had disclosed his history of consistent bedwetting until his 10-year annual examination. He was reluctant to talk about it and stated that he had simply lived with it. Following verbal consent, a 30-minute FCS session addressed restrictions in the urogenital venous and lymphatic systems. He reported no adverse side effects from the treatment. Phone follow-up three days after treatment confirmed that he had not wet the bed since the day of treatment. Follow-up at 11 and 12 years of age confirmed that complete resolution had been sustained since the day of treatment.

Case 2

A post-menarcheal 15-year-old female with nightly ME managed by desmopressin presented with comorbid juvenile psoriatic arthritis and recent onset of anxiety symptoms. The patient stated that she believed both her arthritis and her bedwetting could not be cured but only managed with medication at that point. Following an initial 45-minute FCS session for chronic rib pain and urogenital restrictions, her rib pain resolved, but her enuresis persisted. A second 30-minute FCS session focused on the remaining TPs not evaluated during the first session. Following this second treatment, the bedwetting stopped immediately. Desmopressin was discontinued. She reported no adverse side effects from either treatment. At 10-month follow-up, she had remained consistently dry every night without the use of desmopressin.

Case 3

A post-menarcheal 12-year-old female presented with nightly ME and a history of recurrent urinary tract infections. Previous attempts with a bedwetting alarm had reduced the frequency of wet nights to two to three per week, but the device was uncomfortable to wear, and she discontinued its use. Her bedwetting frequency returned to nightly after cessation of the alarm. The patient appeared embarrassed and reported that she avoided sleepovers with friends to prevent disclosure of her bedwetting. After an initial FCS session focused on the lymphatic and venous systems of the bladder and intestines, her bedwetting frequency decreased to once per week. Due to the practitioner’s ongoing clinical training schedule, a second treatment was deferred for 12 months. During this one-year interval, the family elected not to pursue other medical interventions or pharmacotherapy, reporting that once-weekly bedwetting was a manageable improvement over baseline. Her bedwetting resolved the night after her second FCS treatment session. She reported no adverse side effects from either session. Subsequent follow-ups at eight weeks and 19 months after the second treatment confirmed sustained resolution of her enuresis.

Informed consent was obtained from the legal guardians, and verbal assent was obtained from all three patients for both FCS treatment and publication of this case series.

## Discussion

This case series reports the immediate and sustained resolution of ME following FCS intervention. As demonstrated in Table 1, all three patients experienced cessation of symptoms within 24 hours of targeted treatment, with results sustained at follow-up ranging from 10 to 24 months. This immediacy of cessation is notable, given that adolescent ME is typically characterized by high severity and low rates of spontaneous resolution [2].

**Table 1:** Clinical Characteristics and Sustained Outcomes of Fascial Counterstrain for Persistent Primary Monosymptomatic Enuresis *Sustained Resolution indicates the duration of total dryness from the final treatment date to the most recent follow-up.

Patient	Age/Sex	Baseline Severity	Sessions	Clinical Outcome	Sustained Resolution*
1	10/M	Nightly	1	Immediate cessation	24 Months
2	15/F	Nightly	2	Immediate cessation	10 Months
3	12/F	Nightly	2	Immediate cessation	19 Months

While the anatomical structures listed in Table 2 represent some of the most common primary TPs that were restricted, each case required a unique set of treatments. For example, Case 1 required treatment of only the venous and lymphatic TPs, whereas in Cases 2 and 3, an additional day of assessment and treatment was required.

**Table 2:** Anatomical Targets for Urological Fascial Counterstrain Assessment This table presents key anatomical structures frequently assessed within the Fascial Counterstrain framework; it is not an exhaustive list. Treatment is individualized, targeting only those structures exhibiting clinical restriction.

Nerves	Arteries	Veins/Lymphatics	Viscera	Supporting Fascia
Sympathetic postganglionic nerves and ganglia	Renal arteries	Renal veins	Kidneys (superior/inferior)	Mesentery (lateral/medial)
Parasympathetic pelvic splanchnic nerves	Vesical arteries	Vesical veins	Ureters	Visceral adipose
Vagus nerve	Internal iliac arteries	Internal iliac veins	Bladder	
Pudendal nerve			External urethral sphincter	

In addition to the immediate symptom improvement, another striking common feature among the cases was the persistence of symptom resolution that began the night after a treatment session. Although treatment improvement in these cases was simply by family report without objective measurements, each family’s report included gratitude and disbelief that suddenly the adolescent had only dry nights. With the previously reported unlikelihood of spontaneous regression in this age group, the FCS outcome seems dramatic compared to the mainstay therapies of bedwetting alarms and their training requirements or desmopressin, which requires nightly dosing for continued effect.

The theoretical mechanism of action of FCS is also intriguing in light of the literature that supports an association between inflammatory conditions and ME. In a prospective study, the pro-inflammatory cytokine IL-6 was found to be higher in adolescents with enuresis and in adolescents who had higher adverse childhood event scores [6]. In another study, frequent urinary tract infections and the death of a parent as an adverse childhood event were more strongly correlated with ME than family history and male sex in meta-analysis data [1]. Exploring FCS’s theoretical mechanism of action, which proposes the release of pro-inflammatory cytokines, including IL-6, from localized interstitial spaces, may contribute to the inflammation-related literature on ME. As a simple retrospective study of three cases, these results are limited by small sample size, lack of a control group, and absence of objective measurements beyond family report. Given the paucity of adverse side effects reported and the high patient satisfaction rates, further investigation seems warranted.

## Conclusions

ME that persists into adolescence and adulthood imposes a profound physical and psychosocial burden on millions of individuals. This case series demonstrates that Fascial Counterstrain therapy may have facilitated immediate and sustained clinical resolution in this population, even when conventional treatments had failed. The striking timeline of symptom cessation, if related to FCS, may offer a lower burden of care compared to long-term medication or intensive alarm training. The potential link between localized inflammation and the known inflammatory associations of persistent ME represents a promising avenue for future research into this manual therapy intervention.
